# Comprehensive analysis of an immune-related ceRNA network in identifying a novel lncRNA signature as a prognostic biomarker for hepatocellular carcinoma

**DOI:** 10.18632/aging.203250

**Published:** 2021-07-08

**Authors:** Rui Chen, Yunlong Chen, Wenjie Huang, Yingnan Zhao, Wang Luo, Jinyu Lin, Zhuangxiong Wang, Jian Yang

**Affiliations:** 1 Department of Hepatobiliary Surgery I, General Surgery Center, Zhujiang Hospital, Southern Medical University, Guangzhou, China; 2 Guangdong Provincial Clinical and Engineering Center of Digital Medicine, Guangzhou, China; 3 Institute of Hepatopancreatobiliary Surgery, Chongqing General Hospital, University of Chinese Academy of Sciences, Chongqing, China

**Keywords:** hepatocellular carcinoma (HCC), immune-related competitive endogenous RNA (ceRNA) network, immune-related (long) lncRNA, prognostic signature, immunotherapy

## Abstract

The function of competitive endogenous RNA (ceRNA) network in the immune regulation of hepatocellular carcinoma (HCC) is unclear. Our study aimed to construct an immune-related ceRNA network and develop an immune-related long noncoding RNA (lncRNA) signature to assess the prognosis of HCC patients and to optimize the treatment methods. We firstly constructed a ceRNA regulatory network for HCC using differentially expressed lncRNAs, mRNAs and microRNAs (miRNAs) from the Cancer Genome Atlas. A signature was constructed by 11 immune-related prognostic lncRNAs from the ceRNA network. The survival analysis and receiver operating characteristic analysis validated the reliability of the signature. Multivariate Cox regression analysis revealed that the signature could act an independent prognostic indicator. This signature also showed high association with immune cell infiltration and immune check blockades. LINC00491 was identified as the hub lncRNA in the signature. *In vitro* and *in vivo* evidence demonstrated that silencing of LINC00491 significantly inhibited HCC growth. Finally, 59 lncRNAs, 21 miRNAs, and 26 mRNAs were obtained to build the immune-related ceRNA network for HCC. In conclusion, our novel immune-related lncRNA prognostic signature and the immune-related ceRNA network might provide in-depth insights into tumor-immune interaction of HCC and promote better individual treatment strategies in HCC patients.

## INTRODUCTION

Because of the high rate of incidence, hepatocellular carcinoma (HCC) has been a prevailing malignancy resulting in high mortality, especially in Asian countries that accounts for 75–80% of cases reported globally [1, 2]. Metastasis and recurrence are still the common causes of poor prognosis of HCC patients, even if some therapeutic strategies have been demonstrated to be relatively effective [3]. And the fact that most of HCC patients cannot be diagnosed at an early stage is also related to the poor prognosis [4]. Therefore, thoroughly studying the underlying molecular mechanisms associated with hepatocarcinogenesis is particularly necessary.

In the competitive endogenous RNA (ceRNA) interaction network, lncRNAs acting as endogenous molecular sponges competitively bind to miRNAs via shared miRNA response elements (MRE) with reverse complementary binding seed regions. Indirectly, mRNAs can be affected and manifested with the increased or decreased mRNA expression levels [5]. Recently, the immune system has been shown to have essential effect on the progression of cancer. Besides, the hypothesis that ceRNAs are involved in immune regulation and affecting HCC tumorigenesis and development have been validated by various experiments [6]. Thus, the lncRNAs that regulate the immune microenvironment of HCC have become a hot spot in research.

It is therefore important to carry out a comprehensive analysis of the HCC ceRNA network in aspect of the regulatory functions. The obtained public databases have been widely used to build a lncRNA-miRNA-mRNA ceRNA regulatory network and to mine potential prognostic biomarkers in several types of cancers, such as cholangiocarcinoma, gastric carcinoma, colon adenocarcinoma (CA) and HCC [5, 7]. The established networks are conducive to understand complex interactions of lncRNAs, miRNAs and mRNAs. And the identification of more accurate markers based on the ceRNA network may also contribute to the early diagnosis of cancer, the effectiveness of treatment, and the prognosis of patients. However, there is a lack of studies on the ceRNA network in HCC from the immune regulation viewpoint. Exploring specific ceRNA networks associated with immune regulation might facilitate to understand the tumor-immune interactions, which has great prospects in the therapeutic interventions of HCC. Based on the accumulating evidence, it is shown that ceRNA-related genes do greatly work in the occurrence, development and prognosis of most cancer types [8]. Thus, ceRNA-and-immune-related lncRNAs may also potentially become diagnostic biomarkers or treated targets for HCC. In addition, there is still no study on the construction of prognostic model of HCC by using immune-related lncRNAs from the ceRNA network.

Hence, our study firstly used the Cancer Genome Atlas (TCGA) database to identify differentially expressed (DE) lncRNAs, miRNAs, and mRNAs of HCC. A ceRNA regulatory network of HCC was then constructed by DElnRNAs, DEmiRNAs and DEmRNA. The development of a prognostic signature was based on the identification of the ceRNA-and immune-related lncRNAs. Thereafter, the relationship between the lncRNA signature and immune checkpoint blockades (ICBs) was investigated, the role of a hub gene in the prognostic signature was validated, and an immune-related ceRNA network was further constructed by the ceRNA-and-immune-related DElncRNAs, DEmiRNAs and DEmRNAs. This study might supply some in-depth insights into the tumor-immune interactions in HCC and promote better individual treatment strategies.

## RESULTS

**Figure 1:**
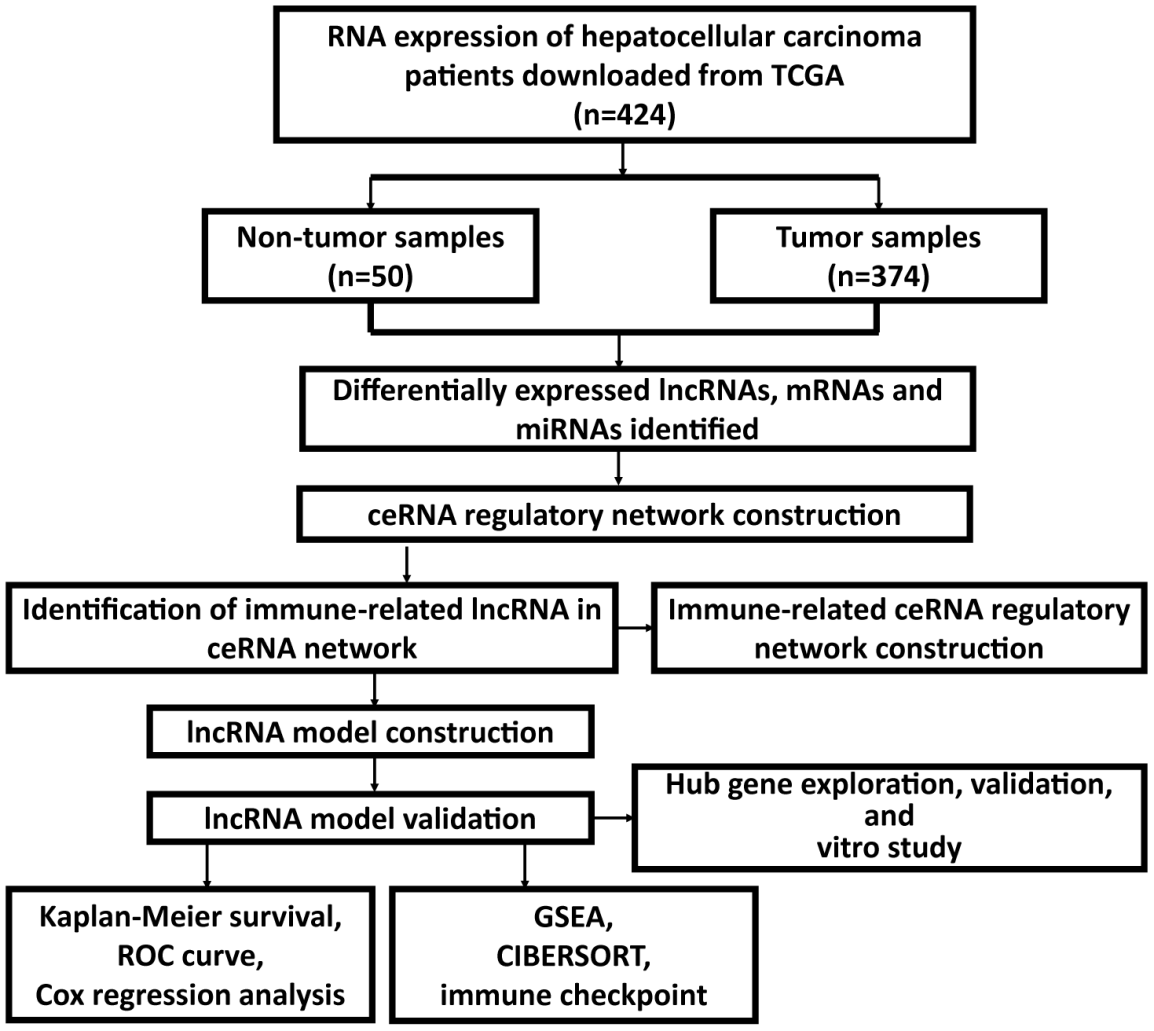
Flow chart of the whole study.

The flow chart of the whole study was presented (Figure 1).

### DElncRNAs, DEmRNAs, and DEmRNAs in HCC

**Figure 2:**
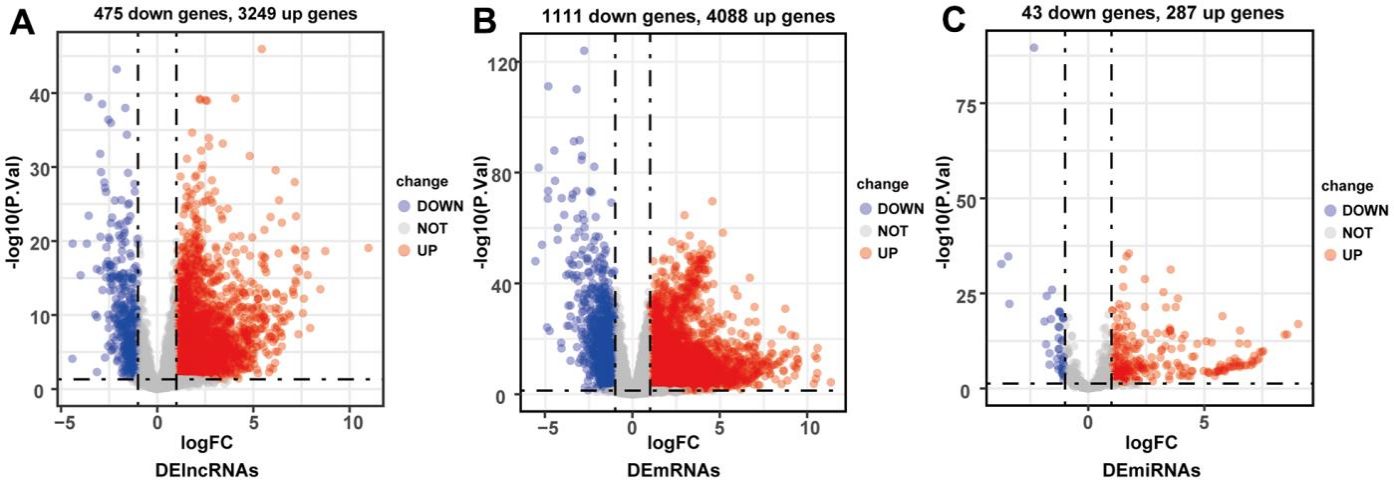
Volcano plots of differentially expressed lncRNAs (**A**), mRNAs (**B**) and miRNAs (**C**). Red plots represented up-regulated genes and green ones represented down-regulated ones. Black plots were genes that did not reach the criteria of differentially expressed genes.

By comparing HCC and adjacent normal liver tissues in TCGA-LIHC database (p < 0.05, |log_2_-fold change (FC)| > 1), a total of 3724 DElncRNAs (3249 upregulated and 475 downregulated), 5199 DEmRNAs (4088 upregulated and 1111 downregulated), and 330 DEmiRNAs (287 upregulated and 43 downregulated) were identified (Figure 2A–2C).

### Constructing the lncRNA-miRNA-mRNA ceRNA network of HCC

The lncRNA-miRNA pairs containing 332 lncRNAs (286 up-regulated and 46 down-regulated) and 30 DEmiRNAs were identified in the miRcode online database. The target gene predictions of 30 DEmiRNAs were identified by using following online tools: TargetScan, miRDB and miRTarBase. The interaction pairs of miRNA–mRNA involving 30 DEmiRNAs and 1494 DEmRNAs were confirmed. The predicted target gene was matched with DEmRNAs, and a total of 242 target DEmRNAs were obtained. After the removal of the 1 remaining miRNA-lncRNA pair, 331 DElncRNAs, 29 DEmiRNAs, and 242 DEmRNAs were finally obtained and the ceRNA network of HCC was built based on them (Supplementary Figure 1 and Supplementary Table 1).

### Constructing and validating the ceRNA-and-immune-related lncRNA prognostic signature

**Figure 3:**
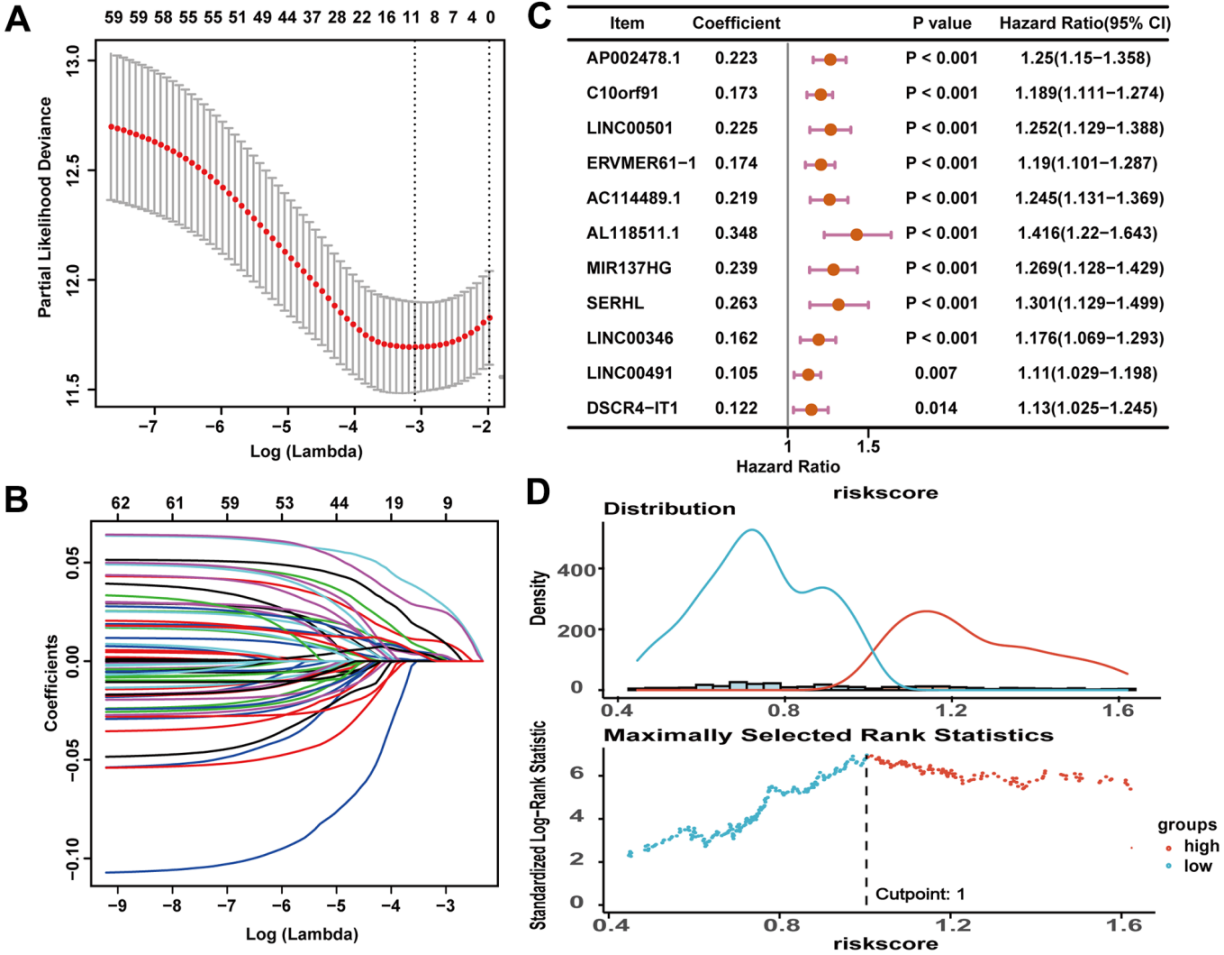
**An 11-lncRNA immune-related signature was established to predict the overall survival of HCC patients.** (**A**) 10-fold cross-validations result identified optimal values of the penalty parameter λ. (**B**) LASSO coefficient profiles of lncRNA with p < 0.05. (**C**) The association between each lncRNA and overall survival. (**D**) The optimal cutoff value of the risk score.

Pearson correlation analysis revealed that 172 lncRNAs out of the 331 ceRNA-related DElncRNAs showed correlation with 1308 immune-related mRNAs (|R| > 0.4 and P < 0.001). The 172 lncRNAs were identified as ceRNA-and-immune-related lncRNAs to construct an immune-related prognostic signature (Supplementary Table 2). In the training cohort, the 172 lncRNAs were subjected to univariate Cox regression analysis and least absolute shrinkage and selection operator (LASSO) Cox regression analyses with log λ ≈ -3.10. Then 11 prognosis-related lncRNAs were filtered out (Figure 3A–3C and Table 1). The risk score of the prognosis signature was calculated as follows:

**Table 1:** The LASSO regression analysis results.

**Gene**	**Coefficient**
AP002478.1	0.102670442
C10orf91	0.055778189
LINC00501	0.035841281
ERVMER61-1	0.008492106
AC114489.1	0.026966520
AL118511.1	0.131909917
MIR137HG	0.048942893
SERHL	0.059437958
LINC00346	0.007189903
LINC00491	0.003028755
DSCR4-IT1	0.005353952

Risk score = (0.102670442*expression value of AP002478.1) + (0.055778189*expression value of C10orf91) + (0.035841281*expression value of LINC00501) + (0.008492106*expression value of ERVMER61-1) + (0.026966520*expression value of AC114489.1) + (0.131909917*expression value of AL118511.1) + (0.048942893*expression value of MIR137HG) + (0.059437958*expression value of SERHL) + (0.007189903*expression value of LINC00346) + (0.003028755*expression value of LINC00491) + (0.005353952*expression value of DSCR4-IT1).

**Figure 4:**
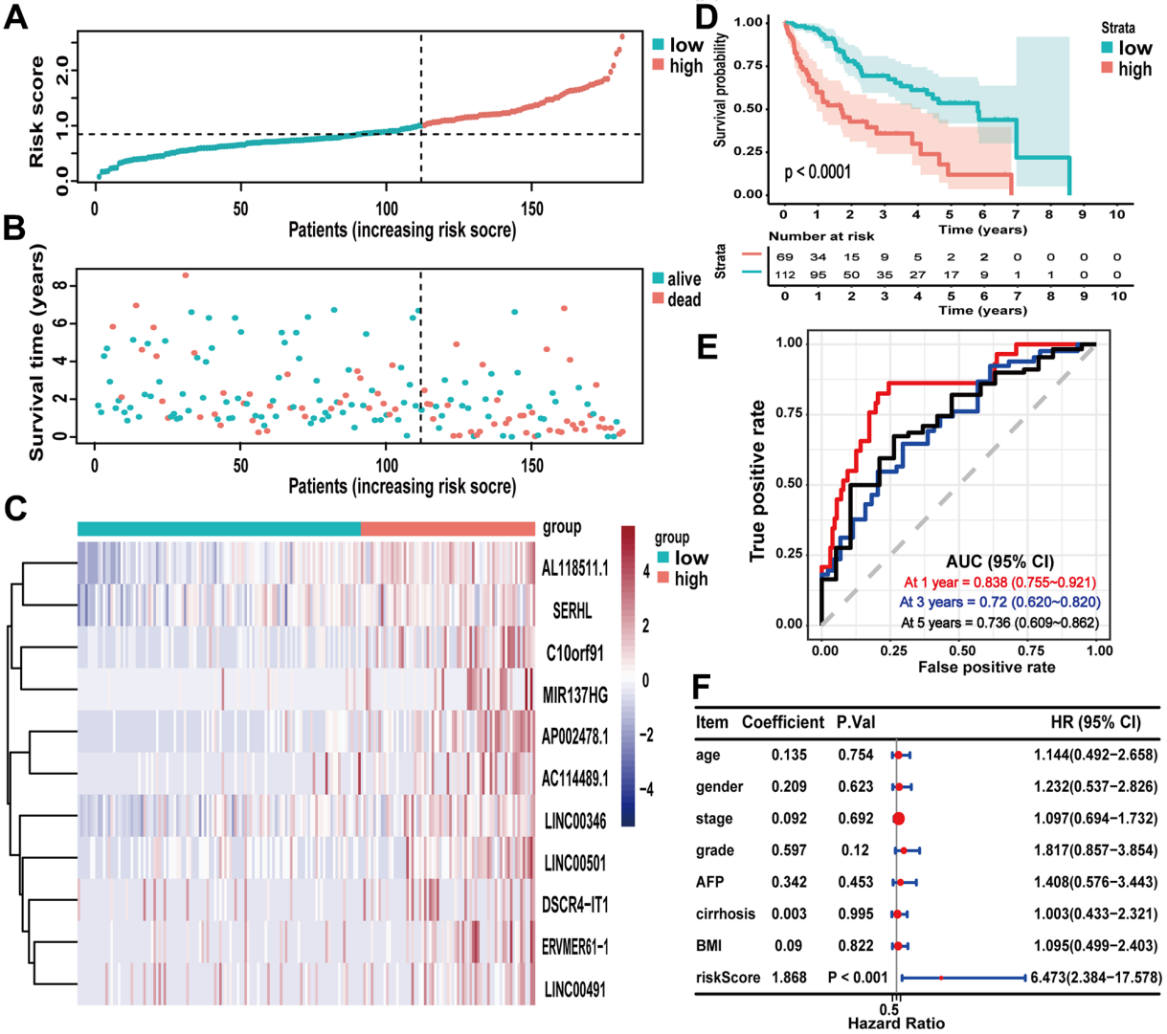
**Evaluating the predictive power of the lncRNA signature in the training cohort.** (**A**–**C**) Distribution of risk score, survival status, and lncRNA expression of patients in the training cohort; (**D**) Kaplan-Meier survival curve of the high-risk and low-risk groups in the training cohort; (**E**) time-dependent ROC curves and AUC based on the training cohort for 1-year, 3-year, and 5-year overall survival; (**F**) forest plot for multivariate Cox regression analysis.

The optimal cutoff value of the risk score in the training cohort, which divided 181 HCC samples in the training cohort into the high-risk group (n = 69) and the low-risk group (n = 112), was 1.00 (Figure 3D). According to the results which were illustrated in Figure 4A, 4B, higher risk scores were associated with more deaths (Figure 4A, 4B). And the 11 lncRNAs expression levels were upregulated in the high-risk group (Figure 4C). According to the Kaplan-Meier survival curve in Figure 4D, the low-risk group had longer survival time than the high-risk group (P < 0.0001). The receiver operating characteristic (ROC) curve suggested that the risk score could predict the 1-year (0.838), 3-year (0.72) and 5-year (0.736) survival rate in HCC patients effectively (Figure 4E). Furthermore, as illustrated in Figure 4F, multivariate Cox regression analyses suggested that the risk score of the signature (HR = 1.951, P < 0.001) could be the independent prognostic indicator of overall survival (OS).

The survival model with the same cutoff value (1.00) of the training cohort was applied to the testing cohort (n = 187) and the entire TCGA-LIHC cohort (n = 368), and the validation results of the signature in these two cohorts also demonstrated excellent capacity for survival prediction (Supplementary Figures 2, 3).

### Correlation of clinical characteristics with the immune-related lncRNA prognostic signature

**Figure 5:**
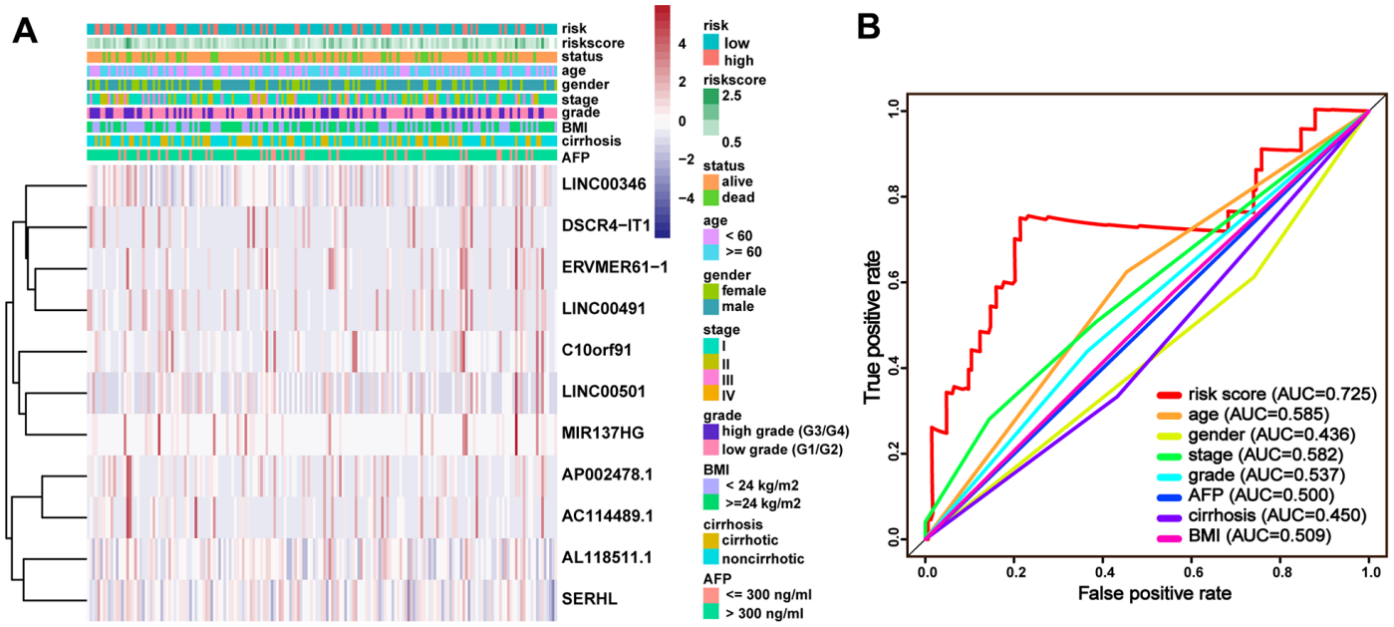
**Clinical characteristics with the lncRNA prognostic signature in the entire TCGA-LIHC cohort.** (**A**) Distribution of clinicopathologic features, and lncRNA expression in the low-risk and high-risk groups; (**B**) time-dependent ROC curves and AUC for 5-year overall survival.

To assess the wide applicability of the signature, the entire TCGA-LIHC cohort was classified into different groups by different clinical characteristics to perform the correlation analysis. The stratification analysis results suggested that the clinical characteristics exhibited distribution patterns were consistent with the risk score of the signature (Figure 5A). The ROC curves for 5-year OS manifested that the risk score (AUC = 0.725) was better than other clinical characteristics (Figure 5B), indicating the considerable reliability of the immune-related lncRNA signature for HCC.

### Cancer hallmark and immune signature analysis

**Figure 6:**
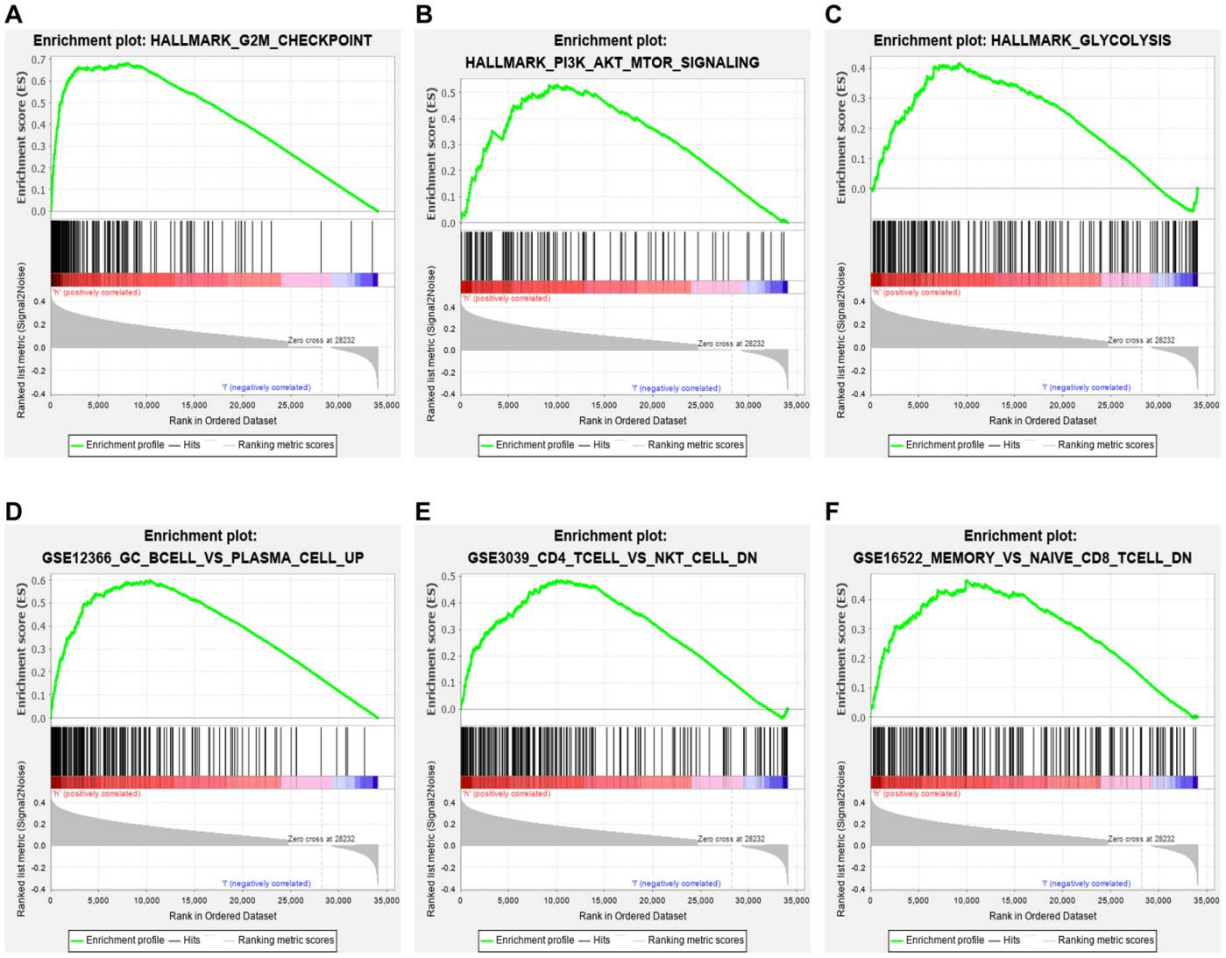
**GSEA.** Hallmark enrichment analysis in this study revealed that the immune-related lncRNA signature in the high-risk group was primarily linked with (**A**) G2M checkpoint, (**B**) PI3K/AKT/MTOR signaling pathway, and (**C**) glycolysis. The immune-related lncRNA model also regulated immunologic signatures within the immune system, such as (**D**) Germinal center (GC) B cell vs. plasma cell up, (**E**) memory vs. naïve CD8 T cell down and (**F**) CD4 T cell vs. NK T cell down.

Hallmark enrichment analysis (h.all.v7.2.symbols) in this study revealed that the high-risk group was primarily linked with G2M checkpoint, PI3K/AKT/MTOR signaling pathway, glycolysis, and some canonical signaling pathways (Figure 6A–6C). These findings might help to understand the biological functions of the ceRNA-and-immune-related signature. In addition, our signature also regulated some immunologic signatures (c7.all.v7.0.symbols), such as germinal center (GC) B cell vs. plasma cell up, memory vs. naïve CD8 T-cell down and CD4 T-cell vs. NK T-cell down, demonstrating that the lncRNA signature might play an essential role in immune-related regulation (Figure 6D–6F).

### Relation of the lncRNA signature with immune cell infiltration and ICB

**Figure 7:**
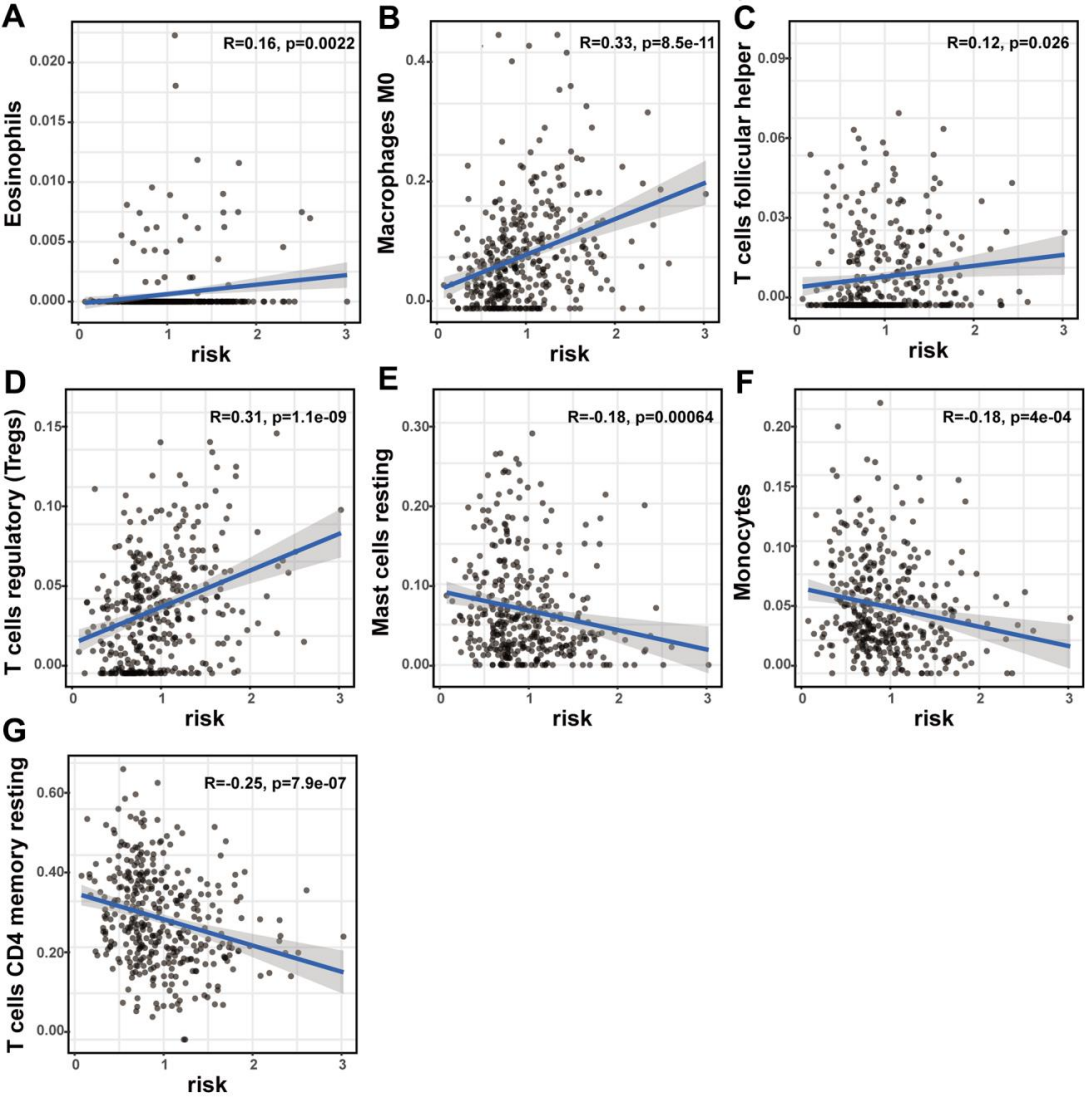
**The correlation of the lncRNA signature with immune cell infiltration and ICB therapy-related genes.** The association of the lncRNA signature and tumor immune cell infiltration. The significant correlation with immune infiltration of (**A**) eosinophils, (**B**) M0 macrophage, (**C**) follicular helper T cells, (**D**) regulatory T cells, (**E**) resting mast cell, (**F**) monocyte and (**G**) CD4 memory resting T cells.

The prognostic lncRNA signature was significantly positively correlated with immune infiltration of eosinophils (R = 0.16; p = 0.0022), M0 macrophage (R = 0.33; p < 0.001), follicular helper T cells (R = 0.12; p = 0.026), and regulatory T cells (R = 0.31; p < 0.001). And the signature was also significantly negatively correlated with immune infiltration of resting mast cell (R = -0.18; p < 0.001), monocytes (R = -0.18; p < 0.001), and CD4 memory resting T cells (R = -0.25; p < 0.001) (Figure 7A–7G). The high correlation between the signature and immune cell infiltration strongly manifested that the signature was consistent with the immunologic signature analysis.

**Figure 8:**
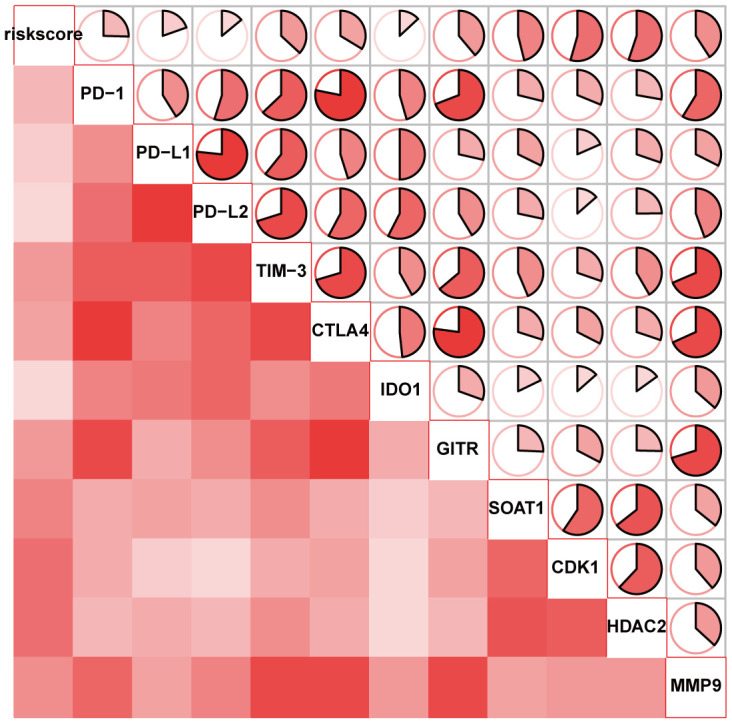
The correlation of the lncRNA signature with ICB therapy-related genes.

The ICB correlation analysis, including PD-1, CTLA4, TIM-3, PD-L1, PD-L2, IDO1, GITR, SOAT1, CDK1, HDAC2 and MMP9, was conducted with the signature. As shown in Figure 8, the lncRNA signature showed significantly positive correlation with all ICBs targeting molecules. This result suggested that the signature might contribute to the evaluation of response to ICB therapy in HCC.

### Identification of the hub gene in the lncRNA signature

LINC00491 has already been recognised as hub oncogene in non-small-cell lung cancer (NSCLC) and CA, but it has never been explored and validated in HCC [9, 10]. Therefore, the role of LINC00491 with regard to HCC requires further assessment.

**Figure 9:**
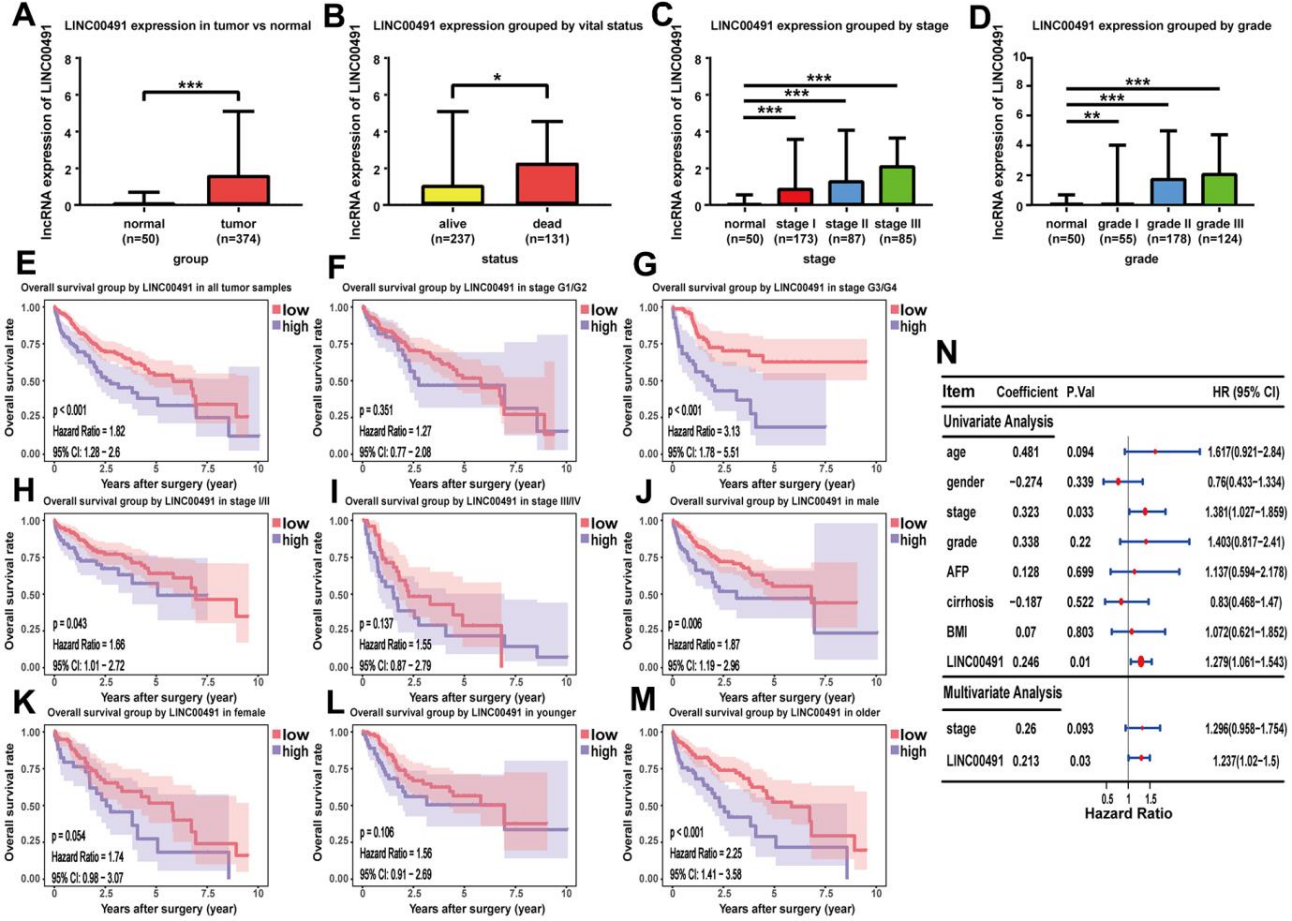
**The characteristic of LINC00491 in TCGA-LIHC.** LINC00491 was significantly differentially expressed in different groups classified by (**A**) HCC tissues, (**B**) vital status, (**C**) AJCC stage and (**D**) histologic grades. (**E**) The potential prognostic ability of LIHC00491 in HCC patients and subgroup analysis of (**F**, **G**) histologic grade, (**H**, **I**) stage, (**J**, **K**) gender and (**L**, **M**) age. (**N**) Forest plot for univariate Cox regression analysis and multivariate Cox regression analysis among LINC00491 and other clinical characteristics. * p < 0.05, ** p < 0.01, *** p < 0.001.

As shown in the boxplot, LINC00491 expression level was significantly upregulated in the HCC tissues, and LINC00491 also significantly differentially expressed in different groups of varying vital status (Figure 9A, 9B). Interestingly, LINC00491 level had the ability to distinguish noncancerous tissues from HCC tissues at AJCC stage I, II, III, and IV (Figure 9C). And LINC00491 could also be applied to distinguish normal tissues from tumor tissues at different histologic grades (Figure 9D). To explore the prognostic ability of LIHC00491, survival analysis found that HCC patients with higher LINC00491 expression level demonstrated a poorer OS (Figure 9E). Subgroup results showed that patients with higher LINC00491 expression level in histologic stage G3/G4, AJCC stage I/II, male and old-age (age ≥ 60) had shorter OS (Figure 9F–9M). Following the multivariate Cox analysis result, LINC00491 expression level could act as an independent prognostic indicator of OS in HCC patients (Figure 9N). Furthermore, LINC00491 was also positively correlated with the above mentioned 11 ICBs (Supplementary Figure 4).

### LINC00491 *in vitro* and in *vivo* experiments

**Figure 10:**
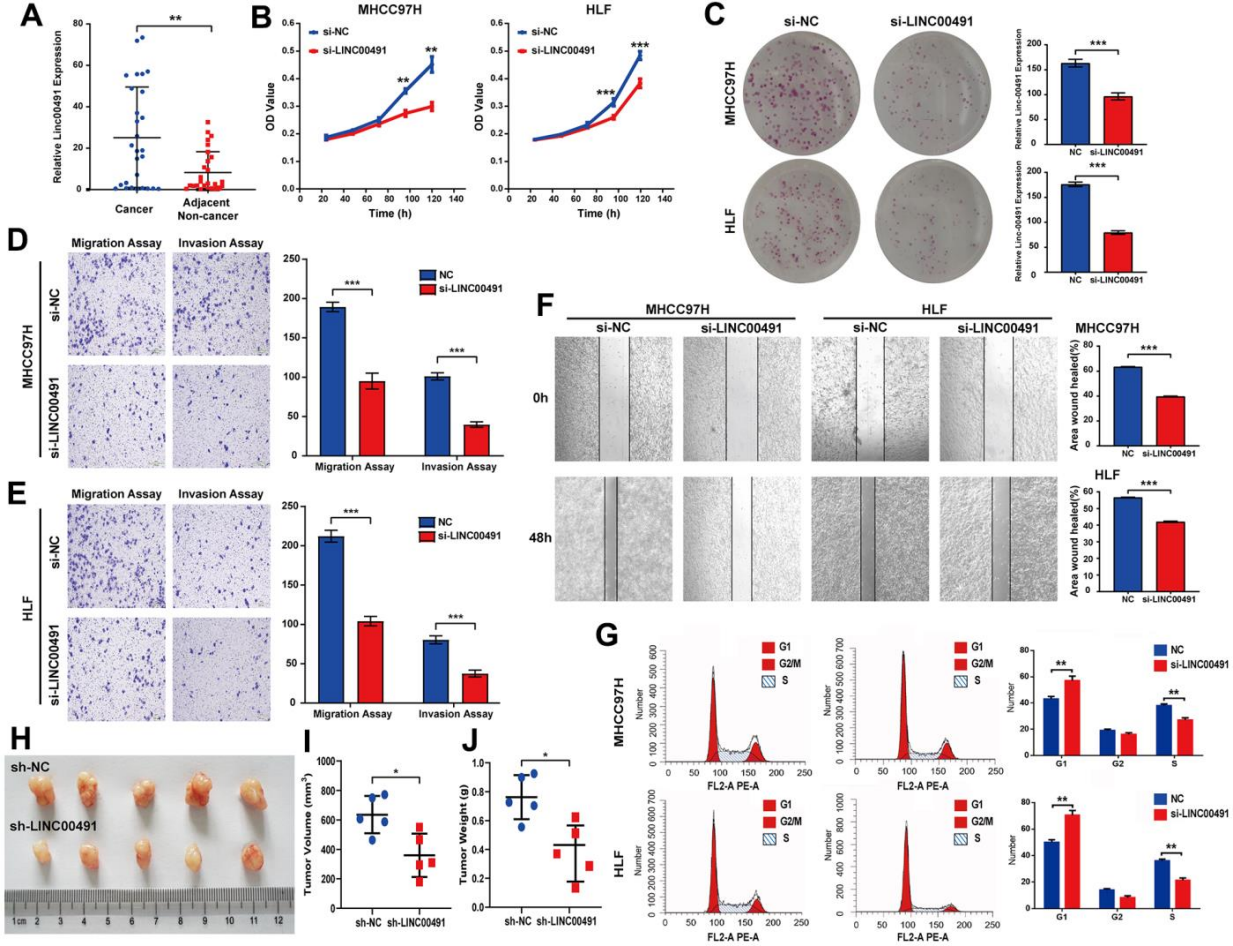
**The role of LINC00491 in HCC.** (**A**) LINC00491 was overexpressed in HCC tissues than that of paracancerous tissues harvested from Zhujiang cohorts. (**B**) The results of CCK-8 assays in the MHCC-97H cell line and the HLF cell line showed lower absorbance in HCC cells with LINC00491 knockdown at 96h and 120h, respectively. (**C**) The colony formation assay indicated that LINC00491 silencing significantly suppressed the growth of HCC cells. (**D**, **E**) The results of transwell assays showed that LINC00491 silencing significantly suppressed invasion and migration rates of HCC cells. (**F**) LINC00491 silencing significantly repressed wound healing in HCC cells. (**G**) The results of cell cycle revealed that LINC00491 depletion significantly inhibited cell cycle progression in MHCC97H and HLF cells. (**H**) Photographs of the subcutaneous tumors are shown. (**I**, **J**) The tumor volumes and weights were measured. * p < 0.05, ** p < 0.01, *** p < 0.001.

LINC00491 was overexpressed in cancerous tissues by qRT-PCR in Zhujiang cohorts, which was consistent with the above database results (Figure 10A). To illuminate the proliferative rate after LINC00491 knockdown in HCC cells, Cell Counting Kit-8 (CCK-8) assays in MHCC-97H and HLF cell lines both showed lower absorbance at 96h and 120h, respectively (Figure 10B). After silencing of LINC00491, the Colony formation assay indicated that the growth of HCC cells was significantly suppressed after silencing of LINC00491 (Figure 10C), transwell assay results showed that the invasion and migration rates of HCC cells were significantly suppressed (Figure 10D, 10E), and wound healing in HCC cells were also significantly inhibited (Figure 10F). Cell cycle progression in MHCC97H and HLF cells revealed that LINC00491 depletion significantly repressed cell cycle progression (Figure 10G). *In vivo* experiments with subcutaneous tumor models were shown in Figure 10H. In accord with *in vitro* observations, tumor volume and tumor weight were both significantly decreased after knockdown of LINC00491 (Figure 10I, 10J).

### Constructing the immune-related ceRNA network in HCC

**Figure 11:**
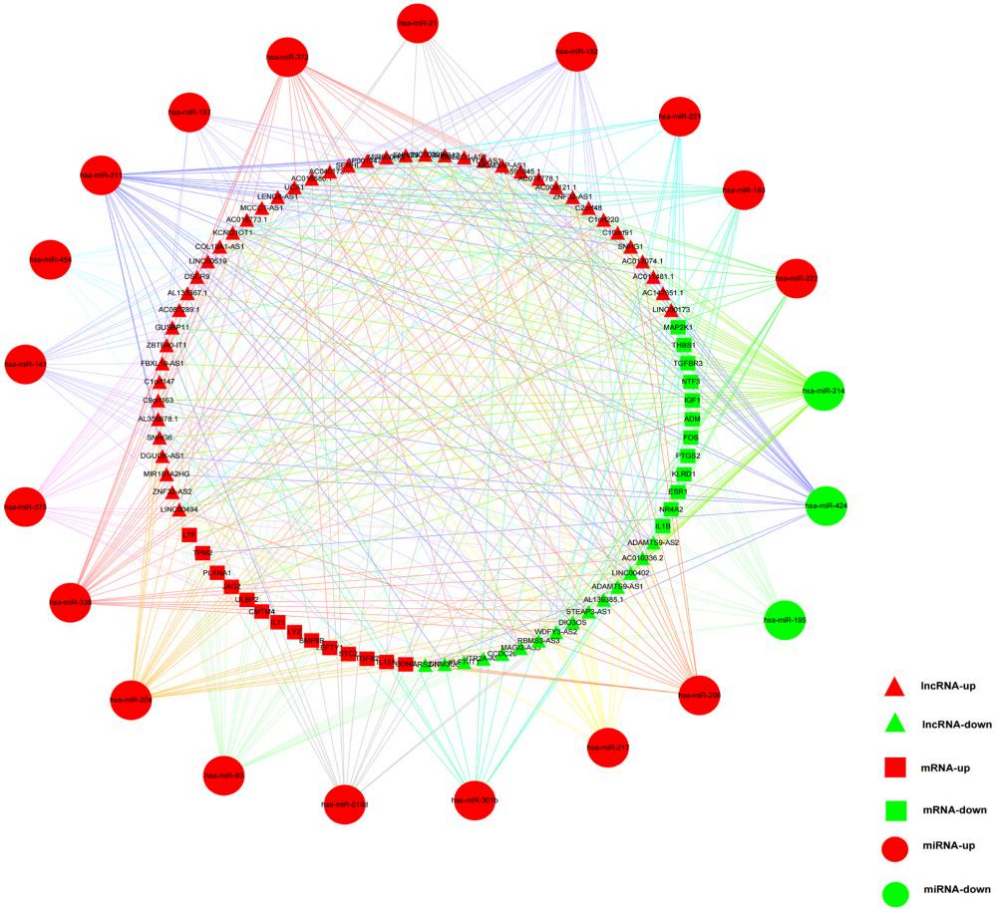
**The immune-related ceRNA regulatory network in HCC.** Red triangles represented upregulated lncRNAs, and green triangles represented downregulated lncRNAs. Red squares and circles stood for upregulated mRNAs and miRNAs, respectively. Green squares and circles represented downregulated mRNAs and miRNAs, respectively.

242 DEmRNAs in the above-mentioned HCC ceRNA network were intersected with the IMMPORT gene list and 33 immune-related DEmRNAs were obtained. To obtain more reliable results, a correlation analysis (R > 0.4, p < 0.001) was carried out between former 172 ceRNA-and-immune-related DElncRNAs and the 33 immune-related DEmRNAs. The results confirmed 161 pairs of lncRNA-mRNA containing 63 lncRNAs and 26 miRNAs. By matching the relationship of the above-mentioned ceRNA network, the immune-related ceRNA network was finally built by 59 lncRNAs, 21 miRNAs, and 26 mRNAs (Figure 11 and Supplementary Table 3).

## DISCUSSION

Elucidating the molecular mechanisms and processes underlying HCC and identifying new potential therapeutic targets would do greatly work in decreasing the mortality of HCC and improving its prognosis. lncRNAs have been demonstrated their essential role in variety of biological processes, such as metabolism, autophagy, inflammation, gene activation, and immune response [11–14].

LncRNAs and miRNAs can modulate cancer immunity and other biological processes through the ceRNA network. In recent years, several ceRNA networks for HCC have been constructed using TCGA database. However, a systematic study on the ceRNA network in HCC from the viewpoint of immune regulation is lacking. Therefore, it might be useful to build an immune-related ceRNA network and comprehensively analyze its regulatory functions.

The construction of the ceRNA network was based on DEG, which largely represented the complexity of tumor growth and metastasis dissemination. In our study, an immune-related ceRNA network including 59 lncRNAs, 21 miRNAs, and 26 mRNAs specific to HCC was developed by matching the relationships of the ceRNA network bulit by DElncRNAs, DEmiRNA and DEmRNAs of HCC. To the best of our knowledge, this was the first study to construct an immune-related ceRNA network in HCC. Exploring specific ceRNA network associated with immune regulation may help to understand tumor-immune interaction and facilitate the discovery of new therapeutic interventions for HCC patients.

In the current study, a ceRNA network was firstly constructed before filtering out immune-related lncRNAs, performing the survival analysis and developing a prognostic signature. This could better identify the potential prognostic biomarker by analyzing the lncRNAs in the ceRNA network. And the following results of the survival analysis, the assessment of predicting efficiency and the correlation analysis with other clinical characteristics strongly manifested the great superiority of the ceRNA-and-immune-related lncRNA signature in HCC, which might provide clinicians with a rationale to use immune targeted therapies in the treatment of HCC patients.

Cancer hallmark and immune signature analysis in our study revealed that the high-risk group of the lncRNA signature largely concentrated in DNA repair, PI3K/AKT/MTOR signaling pathway, glycolysis and immunologic characteristics in HCC. The PI3K/AKT/mTOR signaling pathway does pivotally work in regulating cellular apoptosis, survival, metabolism, and differentiation [15]. We inferred that the 11 lncRNAs might be crucial in regulating HCC through PI3K/AKT/mTOR signaling pathway. Thereafter, the immunologic signature analysis suggested that these lncRNAs had a possible link with immune regulation. Consistent with the previous immune signature analysis, our findings were expanded and confirmed the discovery that the lncRNA signature in HCC showed significant correlation with immune cell infiltration. This suggested that the signature might participate in immune regulation. Nevertheless, the function of immune cell infiltration in HCC still remains unknown. Our preliminary observations provided a perspective opinion to investigate this issue, and further studies were warranted in the future.

Although only less than a third of HCC patients showed significant response to ICB monotherapies, ICB-based strategies including ICB combined with chemotherapy, ICB combined with antiangiogenetic therapy and multiple ICB, might soon be new promising approaches for the treatment of HCC [16, 17]. Thus, identification of biomarkers for predicting ICB immunotherapy responses is considered essential [18, 19]. Some lncRNAs which are significantly associated with immune response can make the treatment prediction of the efficacy to immunotherapy [20, 21]. Interestingly, the signature in our study showed association with immune cell infiltration and it positively correlated with 11 ICBs: PD-1, CTLA4, TIM-3, PD-L1, PD-L2, IDO1, GITR, SOAT1, CDK1, HDAC2 and MMP9. Since these immune checkpoint molecules serve as targets for immunotherapy, the lncRNA signature, which was capable of predicting the characteristics of immune checkpoint inhibition, might have value in clinical setting.

Our study showed that LINC00491 was significantly overexpressed in HCC tissues. And some unfavorable clinical characteristics were also associated with its high expression level. LINC00491 might serve as an independent prognostic indicator of OS. The positive correlation with immune checkpoint genes suggested that LINC00491 may potentially contribute to the measurement in response to HCC ICB immunotherapy. After *in vitro* and *in vivo* experiments, the results were also consistent with the public database analysis. As far as we know, it was the first time that the expression and function of LINC00491 were come up with to be explored in HCC.

There were still some strengths that should be noted in this study after comparing with the existing articles about the examination of the lncRNA prognostic effects on HCC. Firstly, our study included the ceRNA network in HCC from the viewpoint of immune regulation. Exploring the association of the specific ceRNA network with immune regulation might contribute to the understanding of tumor-immune interaction, providing great promise for therapeutic interventions of HCC. Secondly, the prognostic models of HCC were constructed by immune-related lncRNA genes involved in the ceRNA network, making up for the lack of research on prognostic model of HCC.

In conclusion, a novel immune-related 11-lncRNA signature was established, which could be an independent biomarker to predict the prognosis of HCC. And the proposed immune-related ceRNA network might help to clarify the regulatory mechanisms of immune-related lncRNAs as ceRNAs and their contributions in the progression of HCC.

## MATERIALS AND METHODS

### Differential expression analyses

TCGA-LIHC data portal as the HCC database contained 374 HCC-tissue samples and 50 adjacent normal liver-tissue samples. EdgeR package was used to identify DElncRNAs, DEmRNAs and DEmiRNAs between HCC tissues and normal tissues [22]. |log_2_FC| > 1 and p < 0.05 were selected as significant cutoff values. This study does not require the approval of the ethics committee.

### Construction of ceRNA network

The miRcode database was used to predict the relationship between DElncRNAs and DEmiRNAs. The miRDB, miRTarBase, and TargetScan databases were applied to retrieve the target DEmRNAs of DEmiRNAs. Cytoscape software was used to visualize the ceRNA network.

### Identifying immune-related lncRNAs and constructing prognostic signature

The Immunology Database and Analysis Portal (ImmPort) database contained a total of 1811 immune-related genes. Pearson correlation analysis using the thresholds of Spearman's correlation coefficient with an absolute value (|R|) of > 0.4 and p < 0.001 was performed to identify immune-related lncRNAs in the above ceRNA network of HCC.

Based on a 5 : 5 ratio, the entire TCGA-LIHC cohort (n = 368) with prognostic clinical information was randomly divided into the training (n = 181) and the testing (n = 187) cohorts. Univariate Cox regression analysis and LASSO Cox regression analysis were utilized in the training cohort to identify prognostic immune-related lncRNAs and develop a prognostic signature [23, 24]. The optimal cutoff value of risk scores calculated by survminer package (http://cran.r-project.org/) in R software was used to classify the training cohort into the low- and the high-risk groups. And to further confirm the predictive accuracy, the signature was also applied to the testing and the entire TCGA-LIHC cohorts.

### Exploring the bio-function of the lncRNA signature

To find enriched function terms, gene set enrichment analysis (GSEA) with the threshold of p < 0.05 and false discovery rate (FDR) < 0.25 was performed using the Molecular Signatures Database (MSigDB) [25]. CIBERSORT was used to analyze the relation between the immune cell infiltration and the lncRNA signature in HCC samples [26]. To explore the relation with ICB therapy, the correlation analysis was performed between the immune-related signature and 11 immune checkpoint inhibitors, including PD-1, CTLA4, TIM-3, PD-L1, PD-L2, IDO1, GITR, SOAT1, CDK1, HDAC2 and MMP9 [27–35].

### The material preparation of *in vitro* and *in vivo* experiments

28 pairs of fresh frozen specimens with corresponding normal tissues detected by qRT-PCR were obtained from HCC patients of the Department of Hepatobiliary Surgery of Zhujiang Hospital. The Medical Ethics Committee of Zhujiang Hospital had approved our study. According to the manufacturer’s protocol, Lipofectamine 3000 (Invitrogen, Carlsbad, CA, USA) was purchased for transient transfection of LINC00491-Smart Silencer (si-LINC00491) and si-normal control (si-NC) (Ribobio, Guangzhou, China) in MHCC-97H and HLF cell lines. And to conduct animal experiments, the specific short hairpin RNAs to LINC00491 (sh-LINC00491) was used to transfect HLF cells.

### Migration assay

For the transwell assay, cells suspended in serum-free medium were placed in the upper chamber of 24-well Transwell plates (Corning, NY, USA), and the culture medium containing 10% FBS was seeded into the lower chamber. For wound healing assay, the cells were plated into 6-well-plates and cultured in DMEM without FBS. A 200-μl pipette tip was used to scrape the monolayer. At 0 and 48 hours after scratch generation, cell migration was recorded with white light using a phase contrast microscope.

### Proliferation assay, colony formation assay and cell cycle analysis

The CCK-8 assay (Vazyme Biotech, Nanjing, China) was used to determine the proliferative capacity of cells. According to the manufacturer’s instructions, cells were plated in 96-well plates for 4 days, and the absorbance was observed at 450 nm. For colony formation assay, cells were plated in 6-well plate and they were cultured in DMEM containing 10% FBS at 37° C for 2 weeks. Before counting, the colonies were fixed in methanol, and then the colonies were stained with 0.1% crystal violet for 30 min. Cell cycle analysis was carried out as previously described [36].

### Mouse xenograft assay

To perform cells transfection, the left and right backs of nude mice (4-week-old male BALB/c mice) were subcutaneously injected with sh-LINC00491 or sh-NC cells, respectively. The growth of tumors was recorded every 3 days. Mice were killed on the 3 weeks after inoculation, and the resected tumors were weighed for subsequent analysis. All animal experiments were approved by the Ethics Committee for Laboratory Animals of Zhujiang Hospital, Southern Medical University, Guangzhou, China.

## Supplementary Material

Supplementary Figures

Supplementary Table 1

Supplementary Table 2

Supplementary Table 3

## References

[1] Ferlay J, Shin HR, Bray F, Forman D, Mathers C, Parkin DM. Estimates of worldwide burden of cancer in 2008: GLOBOCAN 2008. Int J Cancer. 2010; 127:2893–917. 10.1002/ijc.25516 21351269

[2] Huang Q, Tan Y, Yin P, Ye G, Gao P, Lu X, Wang H, Xu G. Metabolic characterization of hepatocellular carcinoma using nontargeted tissue metabolomics. Cancer Res. 2013; 73:4992–5002. 10.1158/0008-5472.CAN-13-0308 23824744

[3] Chen PJ, Furuse J, Han KH, Hsu C, Lim HY, Moon H, Qin S, Ye SL, Yeoh EM, Yeo W. Issues and controversies of hepatocellular carcinoma-targeted therapy clinical trials in Asia: experts’ opinion. Liver Int. 2010; 30:1427–38. 10.1111/j.1478-3231.2010.02292.x 20557456

[4] Li D, Zhang J, Li J. Role of miRNA sponges in hepatocellular carcinoma. Clin Chim Acta. 2020; 500:10–19. 10.1016/j.cca.2019.09.013 31604064

[5] Bai Y, Long J, Liu Z, Lin J, Huang H, Wang D, Yang X, Miao F, Mao Y, Sang X, Zhao H. Comprehensive analysis of a ceRNA network reveals potential prognostic cytoplasmic lncRNAs involved in HCC progression. J Cell Physiol. 2019; 234:18837–48. 10.1002/jcp.28522 30916406 PMC6618076

[6] Tay Y, Rinn J, Pandolfi PP. The multilayered complexity of ceRNA crosstalk and competition. Nature. 2014; 505:344–52. 10.1038/nature12986 24429633 PMC4113481

[7] Long J, Bai Y, Yang X, Lin J, Yang X, Wang D, He L, Zheng Y, Zhao H. Construction and comprehensive analysis of a ceRNA network to reveal potential prognostic biomarkers for hepatocellular carcinoma. Cancer Cell Int. 2019; 19:90. 10.1186/s12935-019-0817-y 31007608 PMC6458652

[8] Schmitt AM, Chang HY. Long Noncoding RNAs in Cancer Pathways. Cancer Cell. 2016; 29:452–63. 10.1016/j.ccell.2016.03.010 27070700 PMC4831138

[9] Zhang X, Zhao X, Wang Y, Xing L. Long Non-Coding RNA LINC00491 Contributes to the Malignancy of Non-Small-Cell Lung Cancer via Competitively Binding to microRNA-324-5p and Thereby Increasing Specificity Protein 1 Expression. Cancer Manag Res. 2020; 12:6779–93. 10.2147/CMAR.S264681 32821159 PMC7418158

[10] Wan J, Deng D, Wang X, Wang X, Jiang S, Cui R. LINC00491 as a new molecular marker can promote the proliferation, migration and invasion of colon adenocarcinoma cells. Onco Targets Ther. 2019; 12:6471–80. 10.2147/OTT.S201233 31496744 PMC6698166

[11] Carpenter S, Fitzgerald KA. Cytokines and Long Noncoding RNAs. Cold Spring Harb Perspect Biol. 2018; 10:a028589. 10.1101/cshperspect.a028589 28716885 PMC5983188

[12] Frankel LB, Lubas M, Lund AH. Emerging connections between RNA and autophagy. Autophagy. 2017; 13:3–23. 10.1080/15548627.2016.1222992 27715443 PMC5240835

[13] Majidinia M, Yousefi B. Long non-coding RNAs in cancer drug resistance development. DNA Repair (Amst). 2016; 45:25–33. 10.1016/j.dnarep.2016.06.003 27427176

[14] Mathy NW, Chen XM. Long non-coding RNAs (lncRNAs) and their transcriptional control of inflammatory responses. J Biol Chem. 2017; 292:12375–82. 10.1074/jbc.R116.760884 28615453 PMC5535013

[15] Rahmani F, Ziaeemehr A, Shahidsales S, Gharib M, Khazaei M, Ferns GA, Ryzhikov M, Avan A, Hassanian SM. Role of regulatory miRNAs of the PI3K/AKT/mTOR signaling in the pathogenesis of hepatocellular carcinoma. J Cell Physiol. 2020; 235:4146–52. 10.1002/jcp.29333 31663122

[16] Sim HW, Knox J. Hepatocellular carcinoma in the era of immunotherapy. Curr Probl Cancer. 2018; 42:40–48. 10.1016/j.currproblcancer.2017.10.007 29150141

[17] Liu X, Qin S. Immune Checkpoint Inhibitors in Hepatocellular Carcinoma: Opportunities and Challenges. Oncologist. 2019 (Suppl 1); 24:S3–10. 10.1634/theoncologist.2019-IO-S1-s01 30819826 PMC6394775

[18] Mushtaq MU, Papadas A, Pagenkopf A, Flietner E, Morrow Z, Chaudhary SG, Asimakopoulos F. Tumor matrix remodeling and novel immunotherapies: the promise of matrix-derived immune biomarkers. J Immunother Cancer. 2018; 6:65. 10.1186/s40425-018-0376-0 29970158 PMC6029413

[19] Ribas A, Wolchok JD. Cancer immunotherapy using checkpoint blockade. Science. 2018; 359:1350–55. 10.1126/science.aar4060 29567705 PMC7391259

[20] Vishnubalaji R, Shaath H, Elango R, Alajez NM. Noncoding RNAs as potential mediators of resistance to cancer immunotherapy. Semin Cancer Biol. 2020; 65:65–79. 10.1016/j.semcancer.2019.11.006 31733291

[21] Xu J, Shi A, Long Z, Xu L, Liao G, Deng C, Yan M, Xie A, Luo T, Huang J, Xiao Y, Li X. Capturing functional long non-coding RNAs through integrating large-scale causal relations from gene perturbation experiments. EBioMedicine. 2018; 35:369–80. 10.1016/j.ebiom.2018.08.050 30177244 PMC6156711

[22] Robinson MD, McCarthy DJ, Smyth GK. edgeR: a Bioconductor package for differential expression analysis of digital gene expression data. Bioinformatics. 2010; 26:139–40. 10.1093/bioinformatics/btp616 19910308 PMC2796818

[23] Emura T, Matsui S, Chen HY. compound.Cox: Univariate feature selection and compound covariate for predicting survival. Comput Methods Programs Biomed. 2019; 168:21–37. 10.1016/j.cmpb.2018.10.020 30527130

[24] Tibshirani R. The lasso method for variable selection in the Cox model. Stat Med. 1997; 16:385–95. 10.1002/(sici)1097-0258(19970228)16:4<385::aid-sim380>3.0.co;2-3 9044528

[25] Subramanian A, Tamayo P, Mootha VK, Mukherjee S, Ebert BL, Gillette MA, Paulovich A, Pomeroy SL, Golub TR, Lander ES, Mesirov JP. Gene set enrichment analysis: a knowledge-based approach for interpreting genome-wide expression profiles. Proc Natl Acad Sci USA. 2005; 102:15545–50. 10.1073/pnas.0506580102 16199517 PMC1239896

[26] Newman AM, Liu CL, Green MR, Gentles AJ, Feng W, Xu Y, Hoang CD, Diehn M, Alizadeh AA. Robust enumeration of cell subsets from tissue expression profiles. Nat Methods. 2015; 12:453–57. 10.1038/nmeth.3337 25822800 PMC4739640

[27] Joller N, Kuchroo VK. Tim-3, Lag-3, and TIGIT. Curr Top Microbiol Immunol. 2017; 410:127–56. 10.1007/82_2017_62 28900677 PMC5902028

[28] Wu J, Zhu P, Lu T, Du Y, Wang Y, He L, Ye B, Liu B, Yang L, Wang J, Gu Y, Lan J, Hao Y, et al. The long non-coding RNA LncHDAC2 drives the self-renewal of liver cancer stem cells via activation of Hedgehog signaling. J Hepatol. 2019; 70:918–29. 10.1016/j.jhep.2018.12.015 30582981

[29] Zappasodi R, Sirard C, Li Y, Budhu S, Abu-Akeel M, Liu C, Yang X, Zhong H, Newman W, Qi J, Wong P, Schaer D, Koon H, et al. Rational design of anti-GITR-based combination immunotherapy. Nat Med. 2019; 25:759–66. 10.1038/s41591-019-0420-8 31036879 PMC7457830

[30] Jiang Y, Sun A, Zhao Y, Ying W, Sun H, Yang X, Xing B, Sun W, Ren L, Hu B, Li C, Zhang L, Qin G, et al, and Chinese Human Proteome Project (CNHPP) Consortium. Proteomics identifies new therapeutic targets of early-stage hepatocellular carcinoma. Nature. 2019; 567:257–61. 10.1038/s41586-019-0987-8 30814741

[31] Yearley JH, Gibson C, Yu N, Moon C, Murphy E, Juco J, Lunceford J, Cheng J, Chow LQ, Seiwert TY, Handa M, Tomassini JE, McClanahan T. PD-L2 Expression in Human Tumors: Relevance to Anti-PD-1 Therapy in Cancer. Clin Cancer Res. 2017; 23:3158–67. 10.1158/1078-0432.CCR-16-1761 28619999

[32] Keir ME, Butte MJ, Freeman GJ, Sharpe AH. PD-1 and its ligands in tolerance and immunity. Annu Rev Immunol. 2008; 26:677–704. 10.1146/annurev.immunol.26.021607.090331 18173375 PMC10637733

[33] Sun SJ, Wang N, Sun ZW, Chen J, Cui HW. MiR-5692a promotes the invasion and metastasis of hepatocellular carcinoma via MMP9. Eur Rev Med Pharmacol Sci. 2018; 22:4869–78. 10.26355/eurrev_201808_15623 30070322

[34] Zhai L, Ladomersky E, Lenzen A, Nguyen B, Patel R, Lauing KL, Wu M, Wainwright DA. IDO1 in cancer: a Gemini of immune checkpoints. Cell Mol Immunol. 2018; 15:447–57. 10.1038/cmi.2017.143 29375124 PMC6068130

[35] Huang J, Chen P, Liu K, Liu J, Zhou B, Wu R, Peng Q, Liu ZX, Li C, Kroemer G, Lotze M, Zeh H, Kang R, Tang D. CDK1/2/5 inhibition overcomes IFNG-mediated adaptive immune resistance in pancreatic cancer. Gut. 2021; 70:890–99. 10.1136/gutjnl-2019-320441 32816920

[36] Zhang J, Bian Z, Jin G, Liu Y, Li M, Yao S, Zhao J, Feng Y, Wang X, Yin Y, Fei B, Han X, Huang Z. Long non-coding RNA IQCJ-SCHIP1 antisense RNA 1 is downregulated in colorectal cancer and inhibits cell proliferation. Ann Transl Med. 2019; 7:198. 10.21037/atm.2019.04.21 31205916 PMC6545302

