# Supplementary material for: Comprehensive analysis of an immune-related ceRNA network in identifying a novel lncRNA signature as a prognostic biomarker for hepatocellular carcinoma
Source: Aging (Albany NY). 2021 Jul 8;13(13):17607–28. doi: 10.18632/aging.203250 (PMC8312417; doi:10.18632/aging.203250)
Supplement: Supplementary Figures [file aging-13-203250-s001.pdf]

SUPPLEMENTARY FIGURES

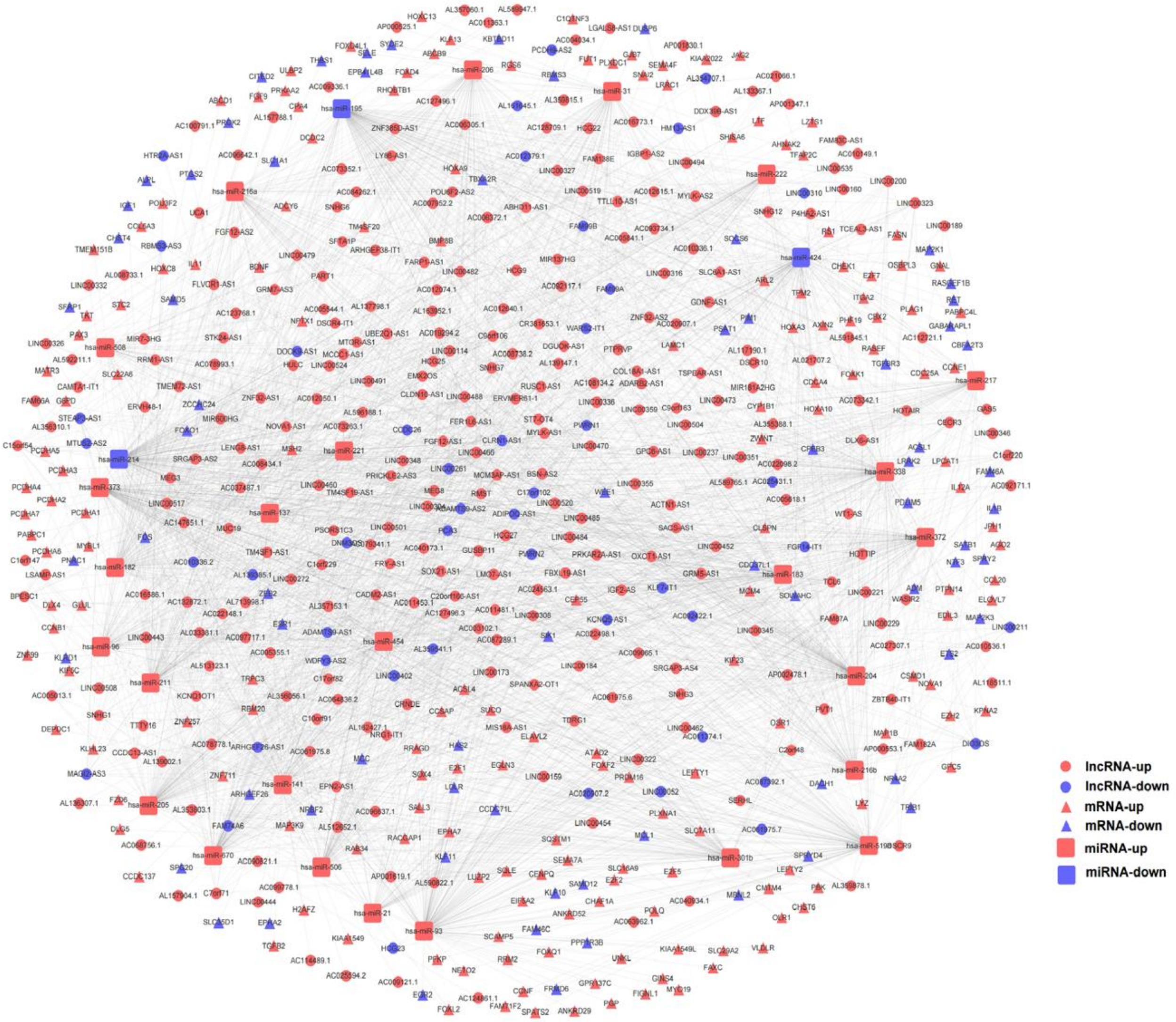

Supplementary Figure 1. The ceRNA regulatory network in HCC. Red circles represented upregulated lncRNAs, and blue circles represented downregulated lncRNAs. Red triangles and squares indicated upregulated mRNAs and miRNAs, respectively. Blue triangles and squares indicated downregulated mRNAs and miRNAs, respectively.

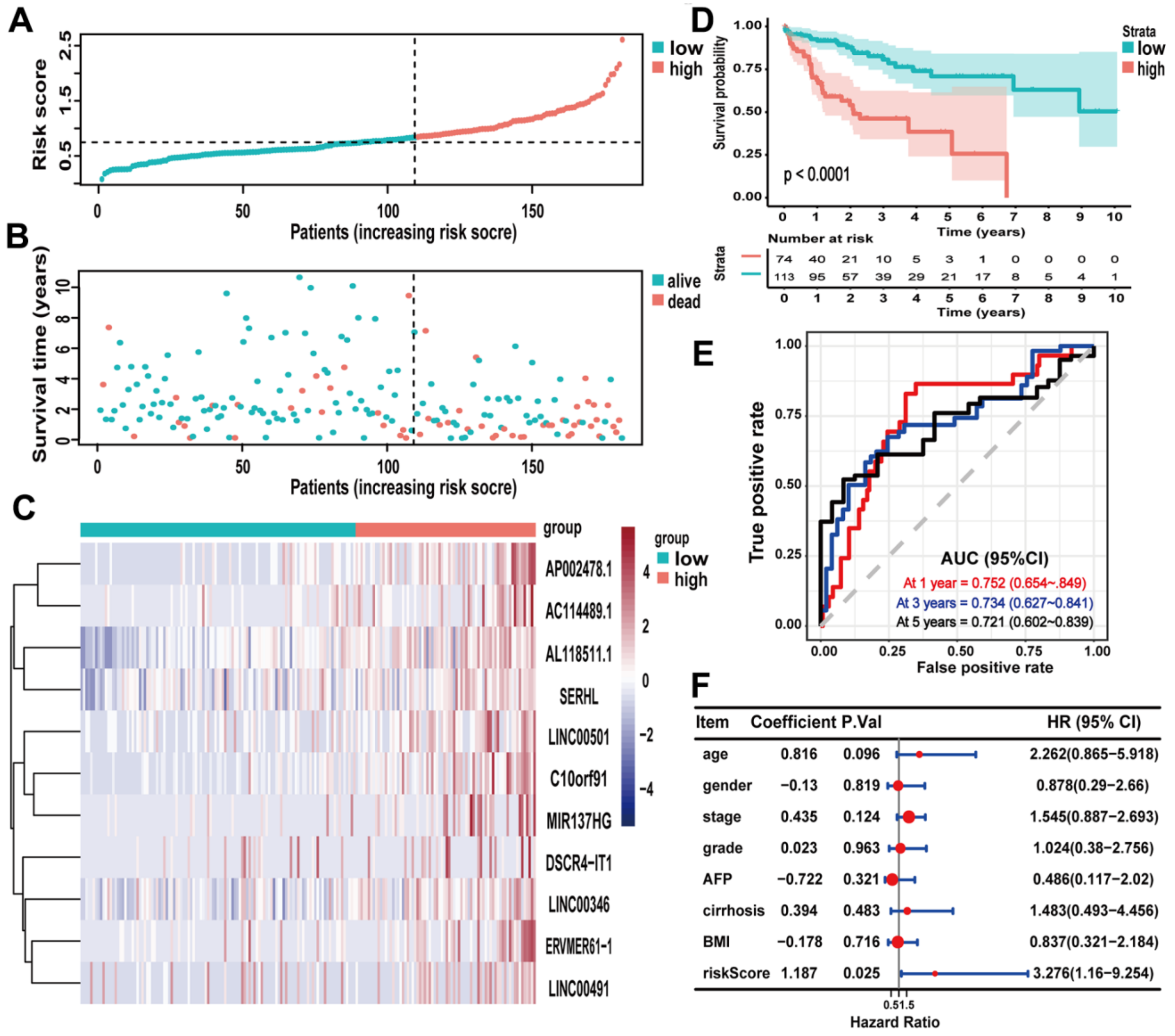

Supplementary Figure 2. Evaluating the predictive power of the lncRNA signature in the testing cohort. (**A**–**C**) Distribution of risk score, survival status, and lncRNA expression of patients in the testing cohort; (**D**) Kaplan-Meier survival curve of the high-risk and low-risk groups in the testing cohort; (**E**) time-dependent ROC curves and AUC based on the testing cohort for 1-year, 3-year, and 5-year overall survival; (**F**) forest plot for multivariate Cox regression analysis.

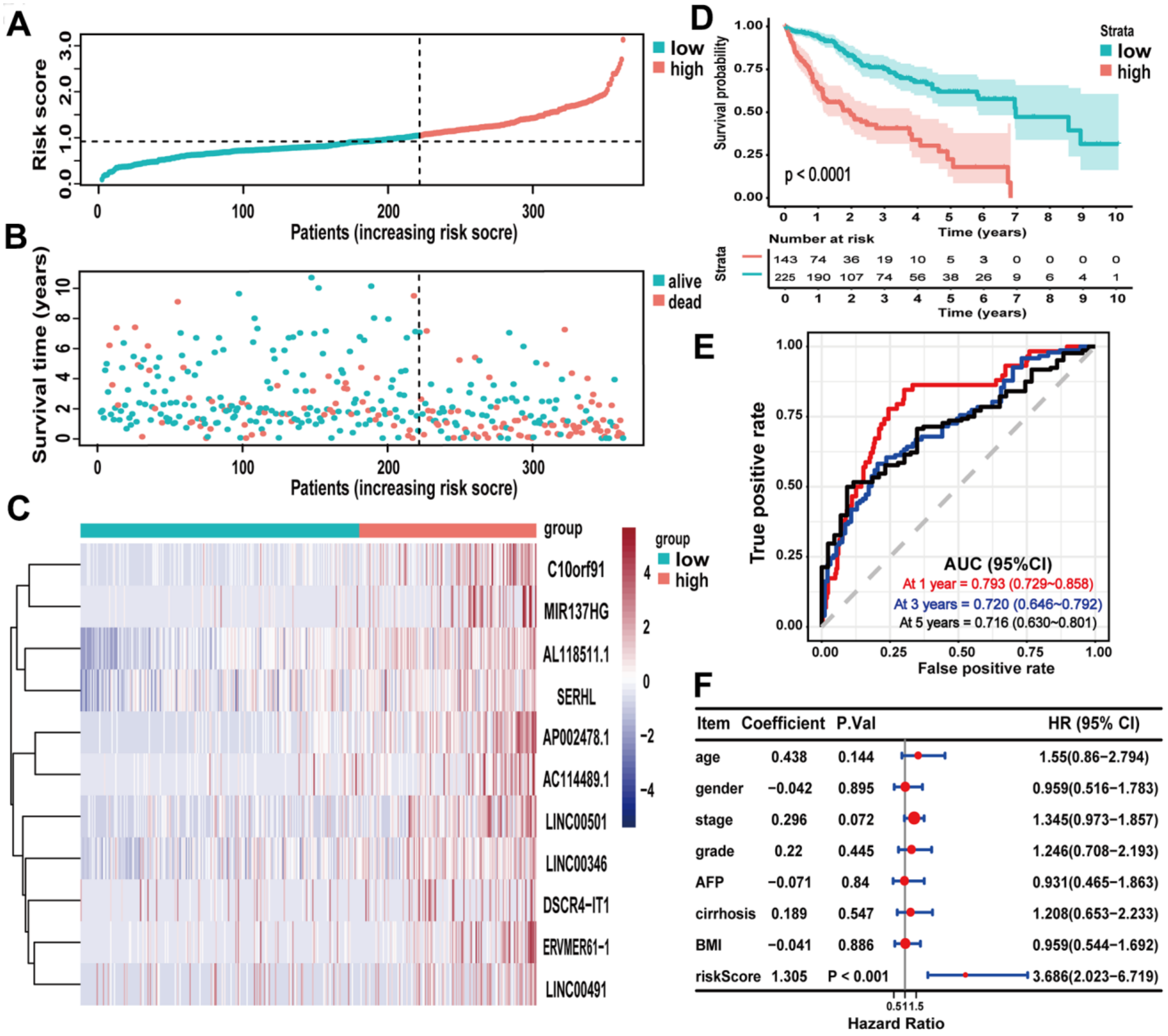

Supplementary Figure 3. Evaluating the predictive power of lncRNA signature in the entire TCGA-LIHC cohort. (**A**–**C**) Distribution of risk score, survival status, and lncRNA expression of patients in the entire TCGA-LIHC cohort; (**D**) Kaplan-Meier survival curve of the high-risk and low-risk groups in the entire TCGA-LIHC cohort; (**E**) time-dependent ROC curves and AUC based on the entire TCGA-LIHC cohort for 1-year, 3-year, and 5-year overall survival; (**F**) forest plot for multivariate Cox regression analysis.

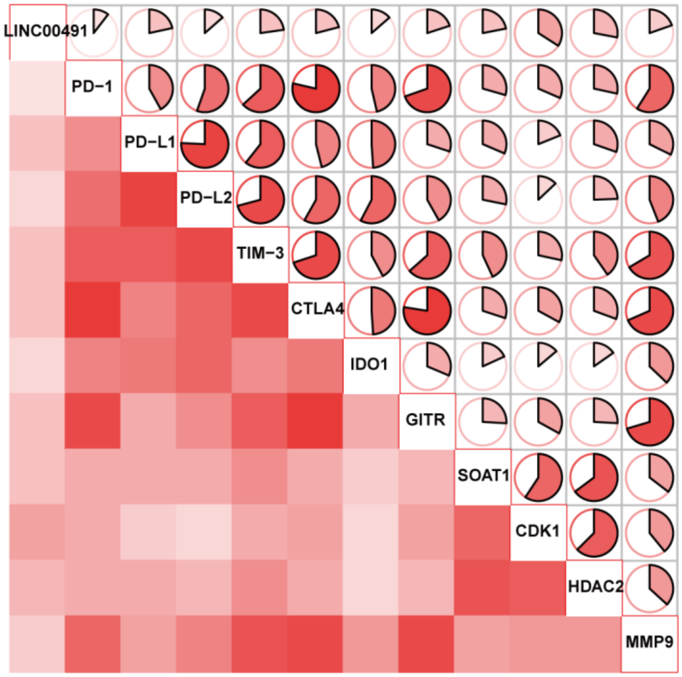

Supplementary Figure 4. The correlation of LINC00491 with ICB therapy-related genes.
