# Supplementary material for: Comprehensive analysis of an immune-related ceRNA network in identifying a novel lncRNA signature as a prognostic biomarker for hepatocellular carcinoma
Source: Aging (Albany NY). 2021 Jul 8;13(13):17607–28. doi: 10.18632/aging.203250 (PMC8312417; doi:10.18632/aging.203250)
Supplement: Supplementary Table 3 [file aging-13-203250-s004.pdf]

Supplementary Table 3. List of genes to build the immune-related ceRNA network in HCC.

59DElncRNA	26DEmRNA	21DEmiRNA
AL591845.1	NR4A2	hsa-miR-93
C1orf147	TPM2	hsa-miR-372
C2orf48	JAG2	hsa-miR-373
SERHL	ESR1	hsa-miR-195
AC087289.1	IL11	hsa-miR-424
AC012074.1	LYZ	hsa-miR-519d
C10orf91	BMP8B	hsa-miR-182
WDFY3-AS2	FOS	hsa-miR-214
COL18A1-AS1	IGF1	hsa-miR-338
C9orf163	TGFBR3	hsa-miR-137
LINC00173	STC2	hsa-miR-141
SNHG12	TGFB2	hsa-miR-204
AC016586.1	THBS1	hsa-miR-211
AL359878.1	BDNF	hsa-miR-21
C1orf220	IL1B	hsa-miR-183
UCA1	LTF	hsa-miR-206
AC010336.2	PLXNA1	hsa-miR-301b
AC011481.1	ULBP2	hsa-miR-454
MCM3AP-AS1	CMTM4	hsa-miR-217
AC016773.1	KLRD1	hsa-miR-221
MIR181A2HG	PTGS2	hsa-miR-222
WARS2-IT1	LEFTY1	
HTR2A-AS1	ADM	
ZNF32-AS1	NTF3	
LINC00323	MAP2K1	
LENG8-AS1	IL12A	
GUSBP11		
CCDC26		
UBE2Q1-AS1		
STEAP3-AS1		
DSCR9		
ZNF32-AS2		
DNM3OS		
MAGI2-AS3		
LINC00402		
LINC00494		
RBMS3-AS3		
MIR600HG		
ZBTB40-IT1		
DGUOK-AS1		
KLF7-IT1		
AC009121.1		
MYLK-AS1		
AP001347.1		
ADAMTS9-AS1		
ADAMTS9-AS2		
AC078778.1		
SNHG3		

MCCC1-AS1
AL133367.1
SNHG6
AC147651.1
SNHG1
AL139385.1
AC040173.1
KCNQ1OT1
DIO3OS
LINC00519
FBXL19-AS1
